# Inhibition of microRNA-200c preserves astrocyte sirtuin-1 and mitofusin-2, and protects against hippocampal neurodegeneration following global cerebral ischemia in mice

**DOI:** 10.3389/fnmol.2022.1014751

**Published:** 2022-11-16

**Authors:** Brian Griffiths, Lijun Xu, Xiaoyun Sun, Majesty Greer, Isabella Murray, Creed Stary

**Affiliations:** ^1^Department of Anesthesiology, Perioperative and Pain Medicine, Stanford University School of Medicine, Stanford, CA, United States; ^2^Howard University College of Medicine, Washington, DC, United States

**Keywords:** glia, stroke, mitochondrial dynamics, SIRT1, MFN2

## Abstract

Memory impairment remains a leading disability in survivors of global cerebral ischemia, occurring secondary to delayed neurodegeneration of hippocampal cornu ammonis-1 (CA1) neurons. MicroRNA-200c (miR-200c) is induced following ischemic stress and we have previously demonstrated that pre-treatment with anti-miR-200c is protective against embolic stroke in mice. In the present study we assessed the role of miR-200c on CA1 neurodegeneration, sirtuin-1 (SIRT1), and mitochondrial dynamic protein expression in a mouse model of transient global cerebral ischemia and *in vitro* in primary mouse astrocyte cultures after simulated ischemia. Mice were subjected to 10 min bilateral common carotid artery occlusion plus hypotension with 5% isoflurane. After 2 h recovery mice were treated with intravenous injection of either anti-miR-200c or mismatch control. Memory function was assessed by Barnes maze at post-injury days 3 and 7. Mice were sacrificed at post-injury day 7 for assessment of brain cell-type specific expression of miR-200c, SIRT1, and the mitochondrial fusion proteins mitofusin-2 (MFN2) and OPA1 *via* complexed fluorescent *in situ* hybridization and fluorescent immunohistochemistry. Global cerebral ischemia induced significant loss of CA1 neurons, impaired memory performance and decreased expression of CA1 SIRT1, MFN2, and OPA1. Post-injury treatment with anti-miR-200c significantly improved survival, prevented CA1 neuronal loss, improved post-injury performance in Barnes maze, and was associated with increased post-injury expression of CA1 SIRT1 and MFN2 in astrocytes. *In vitro*, primary mouse astrocyte cultures pre-treated with miR-200c inhibitor prior to oxygen/glucose deprivation preserved expression of SIRT1 and MFN2, and decreased reactive oxygen species generation, whereas pre-treatment with miR-200c mimic had opposite effects that could be reversed by co-treatment with SIRT1 activator. These results suggest that miR-200c regulates astrocyte mitochondrial homeostasis *via* targeting SIRT1, and that CA1 astrocyte mitochondria and SIRT1 represent potential post-injury therapeutic targets to preserve cognitive function in survivors of global cerebral ischemia.

## Introduction

The hippocampal cornu ammonis-1 (CA1) subregion is central to learning and memory but is selectively vulnerable to ischemic injury. The clinical impact of select CA1 vulnerability to even transient decreases in cerebral blood flow is most commonly represented in survivors of cardiac arrest: while resuscitation rates approach 30% for the over 500,000 cases of adult cardiac arrest in the US per year, over 90% of survivors suffer cognitive impairment (Benjamin et al., 2019) secondary to delayed neuronal loss in CA1. Despite promising pre-clinical trials focused on the delayed nature of CA1 death, no post-injury pharmaceutical interventions are available, and the only effective intervention to prevent CA1 neuronal loss remains application of whole-body hypothermia. To date, the molecular and cellular mechanisms regulating delayed CA1 neuronal loss remain a knowledge gap. We and others have provided increasing evidence supporting a role for microRNAs (miRs) in the brain’s acute and extended response to cerebral ischemia (Ouyang et al., 2013a; Stary and Giffard, 2015; Li and Stary, 2016). MiRs are a well-characterized class of noncoding RNAs that regulate the translation of transcribed genes that can act in a regional and cell-type specific manner.

Astrocytes are the most numerous cell type in the mammalian brain, and support neurons during development, under normal physiologic conditions and after injury (Verkhratsky and Nedergaard, 2018). Astrocytes regulate synaptic glutamate levels, secrete neurotrophic factors, maintain ionic balance, and stabilize neuronal energy balance, all underscored by properly functioning astrocytic mitochondria (Pfrieger, 2009; Heneka et al., 2010). Neurons sustain a high rate of oxidative metabolism while astrocytes preferentially utilize glycolytic pathways, although both cell types contain equivalent numbers of mitochondria (Belanger et al., 2011). Astrocytes also serve to mitigate the downstream effects of pro-inflammatory cytokines on generation of reactive oxygen species (ROS) and secrete soluble factors to indirectly modulate resident neuronal mitochondrial function. Mitochondria are central to normal physiologic brain function and in repair of the injured brain by maintaining phosphorylation potential (ATP) to support energy requirements for a host of biological processes. Mitochondria are well-known targets of ischemic injury and prior work using has demonstrated efficacy in neuroprotection against stroke when astrocyte mitochondria are protected (Xu et al., 2010). A detailed determination of the mechanisms that regulate cell-type specific mitochondrial dysfunction in neurons and astrocytes has been a central barrier impeding novel stroke therapies. Mitochondrial homeostasis is maintained by an interdependent balance between mitochondrial dynamics (fission and fusion), ROS and metabolic energy state (Dorn and Kitsis, 2015; Zhou et al., 2021). Mitochondria maintain a steady state of continuous fusion/fission maintaining the normal physiological function (Dorn and Kitsis, 2015). An imbalance in mitochondrial dynamics can affect energy metabolism and post-stroke neuronal function by regulating the function of mitochondria. Prior work identifies hippocampal subregional and cell-type-dependent states of mitochondrial impairment, underscoring a previously unexploited therapeutic niche. All hippocampal mitochondria are disrupted 2 h after transient global cerebral ischemia but astrocytes exhibit rapid restoration by 24 h (Kumar et al., 2016). Conversely CA1 neuronal mitochondrial function remains selectively disrupted for an extended period of days (Shiino et al., 1998; Kumar et al., 2016) in contrast to neurons in the nearby ischemia-resistant dentate gyrus (DG). The molecular mechanisms regulating this hippocampal subregional- and cell-type specific mitochondrial response are entirely unknown.

MicroRNA-200c (miR-200c) is known to target the mitochondrial regulatory protein sirtuin-1 (SIRT1), which plays a central role in maintaining mitochondrial bioenergetics, fission/fusion balance, and ROS production. We have recently reported that miR-200c selectively increases in astrocytes relative to neurons after cerebral ischemia (Arvola et al., 2021) and demonstrated that miR-200c inhibition protected neurons from experimental stroke (Stary et al., 2015), however, whether miR-200c plays a role in delayed CA1 neuronal cell death after global cerebral ischemia is unknown. Therefore, in the present study we assessed whether post-injury treatment with anti-miR-200c reduced CA1 neurodegeneration in a mouse model of global cerebral ischemia. In parallel we assessed the role of miR-200c in cell-type specific post-injury changes in CA1 SIRT1 and mitochondrial fusion proteins *in vivo* and in primary mouse astrocyte cell cultures.

## Materials and methods

### *In vivo* global cerebral ischemia

All experimental protocols using animals were performed according to protocols approved by the Stanford University Animal Care and Use Committee and in accordance with the National Institutes of Health *Guide for the Care and Use of Laboratory Animals*. Global cerebral ischemia was induced in 8–10 week-old male C57BL/6J mice (Charles River Laboratories, Wilmington, VA, USA) *via* bilateral carotid artery occlusion (two-vessel occlusion, 2VO) + isoflurane (Onken et al., 2012; Kristian and Hu, 2013; Owens et al., 2015). Briefly, after anesthesia induction with 2.5% isoflurane and surgical incisions were complete, hypotension (mean arterial pressure, <40 mmHg) was induced with 5% isoflurane during continuous femoral arterial blood pressure monitoring (TA100 transducer, Moor Instruments, Wilmington, DE, USA). After 2 min of 5% isoflurane the common carotid arteries were clamped bilaterally. After 6 mins of simultaneous 5% isoflurane and bilateral common carotid artery clamping the isoflurane was reduced to 2.5%. Rectal temperature (37 ± 0.5°C) was controlled by a homeothermic blanket (Harvard Apparatus, Cambridge, MA, USA). Respiratory rate, heart rate, and pulse oximetry were monitored with a small animal oximeter (STARR Life Sciences, Oakmount, PA, USA). Cerebral perfusion pressure was continuously monitored *via* laser doppler (Model VMS-LDF1, Moor Instruments). After 10 min clamps were removed and isoflurane was decreased to 1% maintenance until closure of surgical incisions. Core body temperature was maintained during recovery at 37°C with a heating pad post-surgery to eliminate any neuroprotective effects of hypothermia. Mice were then randomized by coin flip and 2 h after global cerebral ischemia mice were re-anesthetized with 2% isoflurane and either anti-miR-200c inhibitor (Anti-miR™ miRNA Inhibitor, #AM17000, ThermoFisher Scientific, Waltham, MA, USA) or mismatch control (MM-control, ThermoFisher Scientific) in sterile saline (100 μl) was administered into the internal jugular vein as previously described (Xu et al., 2015).

### Barnes maze

Memory testing in mice was performed as we have previously reported (Griffiths et al., 2019b). Mice were placed in the center of a circle platform with 20 equally spaced holes and visual clues; one of the holes was connected to a safe chamber (SD Instruments, San Diego, CA, USA). Aversive noise (85 dB) in conjunction with bright light (200 W) was shed on the platform to encourage the mouse to find the safe target. All mice were trained for 4 consecutive days with 3 min per trial, 4 trials per day. Their reference memory was tested on day 3 (short-term retention) and day 7 (long-term retention). Each mouse had only one trial on each of these two test days. The latency to find the target box during each trial was recorded and analyzed in real time by TopScan™ software (CleverSyS, Reston, VA, USA).

### *In vivo* histological assessment

Animals were sacrificed at 7 days after injury by isoflurane overdose, and brains immediately perfused with transcardial ice-cold saline, then fixed with 4% phosphate-buffered paraformaldehyde (PFA) for stereological analysis. Coronal vibratome sections (50 μm) were used for combined immunohistochemical (IHC) analysis and fluorescent *in situ* hybridization (FISH) for miR-200c using miRCURY LNA miR Detection Probes (ThermoFisher Scientific) as we have previously done (Arvola et al., 2019). All fixed sections were stained for miR-200c, the astrocyte marker glial fibrillary acidic protein (GFAP, #ab90601, Abcam, Boston, MA, USA, 1:500 dilution), the mature neuronal marker NeuN (#ab104224, Abcam, 1:500 dilution), SIRT1 (# ab189494, Abcam, 1:500 dilution) and the mitochondrial fusion markers MFN2 (# bs2988R, BiossUSA, Woburn, MA, USA, 1:500 dilution) and OPA1 (#ab42364, Abcam, 1:500 dilution). The CA1 and DG regions were identified anatomically with DAPI and demarcated according to Newton et al. (2005) and as we have previously done (Stary et al., 2016; Griffiths et al., 2019a,b). Images were acquired by an observer blinded to conditions using an upright Zeiss Axio-Imager M2 fluorescent microscope equipped with Apotome 2.0 for optical sectioning, and Zeiss EC-Plan Neofluar 20×, Zeiss Plan Apochromat 40×, and Zeiss LC-Plan Neofluar 63× objectives. Qualitative protein expression with GFAP+ co-localized was performed using the “masking” function in ImageJ v1.49b software (NIH, Bethesda, MD, USA). In brief, Z-stack images were first independently analyzed in the GFAP channel, and then masked to generate GFAP+ regions of interest (ROIs). Next, GFAP+ ROIs were superimposed on the channel of interest, and then fluorescence intensities were measured and collated, for all sections. All imaging for a given protein were collected using a fixed excitation intensity, exposure time, and gain, to minimize variability. No post-imaging processing was performed. An observer blinded to conditions quantified from maximum projection Z-stack images the cell-type specific relative intensity of miR-200c fluorescence and the relative fluorescent intensity of NeuN, mitofusin-2 (MFN2), OPA1, and SIRT1 proteins using StereoInvestigator™ (MicroBrightField, Williston, VT, USA) software and ImageJ v1.49b software (NIH, Bethesda, MD, USA) as we have previously done (Arvola et al., 2019, 2021; Griffiths et al., 2019a).

### Primary brain cell cultures

Primary astrocyte cultures were prepared from postnatal (days 1–3) Swiss Webster mice (Charles River Laboratories, Wilmington, MA, USA) as described previously (Li et al., 2021). Isolated astrocytes were seeded on 24-well plates in plating medium consisting of Eagle’s Minimal Essential Medium (Gibco, Grand Island, NY, USA) supplemented with 10% fetal bovine serum and 10% equine serum (HyClone, Logan, UT, USA), 21 mM (final concentration) glucose, and 10 ng/ml epidermal growth factor. Cultures were maintained at 37°C in a 5% CO_2_ incubator. Primary astrocyte cultures were transfected with 50 nmol negative control (*mir*Vana^®^ #4464061, ThermoFisher Scientific), miR-200c mimic (*mir*Vana^®^ #4464066, ThermoFisher Scientific), or inhibitor (*mir*Vana^®^ #4464084, ThermoFisher Scientific) using Lipofectamine 2000 (Invitrogen) according to the manufacturer’s protocol on day *in vitro* (DIV) 16. In some experiments cultures were co-treated with the SIRT1 activator YK 3-237 (10 μM, Tocris #5667). In parallel experiments astrocytes were selectively cultured from CA1 and DG as previously described (Stary et al., 2016). Briefly, the left and right hippocampi were identified morphologically and by anatomical location, and dissected free in their entirety, while maintaining the anatomical orientation. The dorsal region of the hippocampus containing primarily CA1 was dissected free of the remainder of the hippocampus. The ventral hippocampus (containing DG) was further dissected with removal of the CA3 region. CA1 and DG hippocampal regions from individual animals were pooled, treated with 0.05% trypsin/EDTA (Life Technologies, Carlsbad, CA, USA), and plated in Dulbecco’s modified Eagle medium (Gibco, Grand Island, NY, USA) with 10% equine serum (ES, HyClone), 10% fetal bovine serum (FBS, HyClone) and 10 ng/ml epidermal growth factor (Sigma Chemicals, St Louis, MO, USA). In some experiments primary neuronal cultures were utilized, prepared as previously described (Newton et al., 2005) from embryonic (E16-E18) mouse cortices. Briefly, the dissected cortices were dissociated with 0.05% trypsin/EDTA for 15 min at 37°C, triturated, then plated in medium containing 5% FBS and 5% ES (HyClone). A relatively pure neuronal culture was obtained by adding cytosine arabinoside (3 mol/L, Sigma) 24 h after plating to curb glial proliferation. For all experiments 3–4 independent cultures were tested as replicates within each experiment.

### *In vitro* injury and fluorescent imaging

*In vitro* ischemia was induced by oxygen-glucose deprivation (OGD) as previously described (Ouyang et al., 2011). Briefly, 24 h following transfection primary astrocyte cultures were washed three times with glucose-free culture medium equilibrated with 100% N_2_ in an anoxia chamber maintained at <350 ppm (<0.02%) O_2_ (COY Laboratory Product Inc.). After 3 h OGD the medium was reoxygenated and glucose was added at 5.5 mM. For oxidative stress assays, glucose deprivation alone was selected as an ischemia-like stress for astrocyte cultures, as it reliably induces an extended period of mitochondrial dysfunction with increased ROS production prior to cell death, as we have employed previously (Stary et al., 2016). Cells were maintained at 37°C and 5% CO_2_ in an atmospherically controlled chamber (Ibidi GmbH, Martinsried, Germany) for imaging of live-cell ROS generation after 30 min incubation with 5 μM CellROX™ Green (ThermoFisher Scientific, Waltham, MA, USA). Automated fluorescent image capture was performed at 200× using a Lumascope™ 720 (Etaluma, Carlsbad, CA, USA) as we have previously performed (Xia et al., 2018). For each well five replicate images were obtained and averaged. Mean intensity of fluorescence was quantified by an observer blinded to conditions ImageJ v1.49b software (NIH). Unbiased changes in fluorescence were normalized to MM-control transfection treatment. For assessment of astrocyte SIRT1 and MFN2, fluorescence immunocytochemistry was performed on astrocyte cell cultures in 24-well plates as described previously (Ouyang et al., 2013b). Cultures were fixed in 4% paraformaldehyde for 30 min at room temperature. Nonspecific binding was blocked with 5% normal goat serum and 0.3% Triton X-100 in PBS for 1 h. Cells were incubated with mouse monoclonal primary antibody to SIRT1 (# ab189494, Abcam, 1:100 dilution) or MFN2 (# bs2988R, BiossUSA, 1:100 dilution) overnight at 4°C. Cells were then washed and incubated with Alexa Fluor 488 nm-conjugated secondary antibody (1:500; Invitrogen, Grand Island, NY, USA) for 1 h. Cells were counterstained with the nuclear dye DAPI (4′,6′-diamidino-2-phenylindole, 0.5 μg/ml; Sigma-Aldrich, St Louis, MO, USA) and automated fluorescent image capture was performed at 200× using a Lumascope™ 720 (Etaluma, Carlsbad, CA, USA). For each well nine replicate images were obtained and averaged. Unbiased mean intensity of fluorescence was quantified by an observer blinded to treatment groups using ImageJ v1.49b software (NIH) and normalized to cell count (DAPI). Differences in fluorescence intensity between treatment groups were compared by normalizing to miR-200c mimic wash treatment for each condition.

### Reverse transcription quantitative polymerase chain reaction

Total RNA was isolated with TRIzol^®^ (ThermoFisher Scientific, Waltham, MA, USA) from CA1 tissue 24 h after injury and from astrocyte culture 3 h after wash control or OGD injury. Reverse transcription was performed as previously described (Stary et al., 2015) using the TaqMan MicroRNA Reverse Transcription Kit (Applied Biosystems, Foster City, CA, USA). Predesigned primer/probes for PCR were obtained from ThermoFisher Scientific for mmu-miR-200c–3p (#4426961) and U6 small nuclear RNA (U6, #01973). PCR reactions were conducted as previously described (Stary et al., 2015) using the TaqMan^®^ Assay Kit (Applied Biosystems). Measurements for miR-200c were normalized to U6 (ΔCt) and comparisons were calculated as the inverse log of the ΔΔCT from controls (Livak and Schmittgen, 2001).

#### Immunoblots

Total protein from primary astrocyte cultures was isolated as previously described (Stary et al., 2017). Briefly, cultures were first washed with cold 0.1% phosphate buffered saline, then total cellular protein was quantified by Pierce BCA protein assay kit [ThermoFisher Scientific (Stary et al., 2017)]. Equal amounts of protein were loaded and separated on 10–12.5% polyacrylamide gels, then transferred to Immobilon polyvinylidene fluoride membranes (EMD Millipore Corp). Membranes were blocked with 5% skimmed dry milk and incubated overnight with primary antibody against SIRT1 (Abcam, #ab110304), MFN2 (Abcam, #ab124773), β-actin (LI-COR Bioscience #926–42,210) and/or β-tubulin (Abcam, #ab6046). Membranes were then washed and incubated with secondary antibodies (LI-COR Bioscience) for 1 h followed by washing again and visualizing by using the LICOR Odyssey infrared imaging system. Densitometric analysis of bands was performed *via* Image Studio Lite (LI-COR Biosciences), and the intensity of all proteins was normalized to β-actin or β-tubulin as a control.

### Statistical analyses

Numbers of animals are indicated in figure legends. Data reported are means ± SE. Statistical difference was determined using *t*-test for comparison of two groups or ANOVA followed by Bonferroni correction for experiments with *N* > 2 groups using Sigmaplot (Systat Software, San Jose, CA, USA). *p* < 0.05 was considered significant.

## Results

### Anti-microRNA-200c treatment improves physiologic outcomes after global cerebral ischemia

**FIGURE 1:**
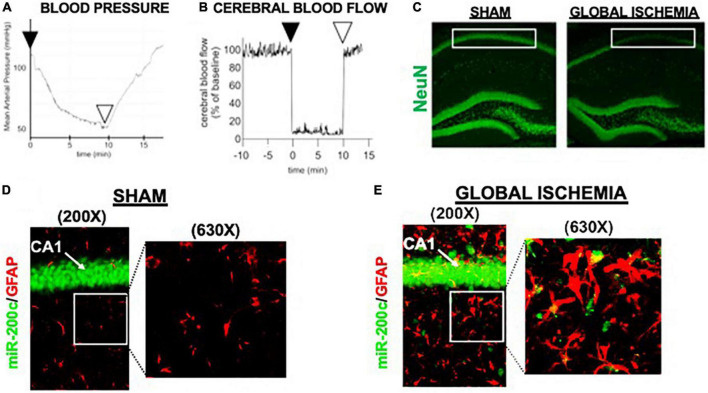
Mouse global cerebral ischemia model. Invasive blood pressure monitoring **(A)** and laser doppler cerebral blood flow measurement **(B)** during bilateral carotid artery occlusion (2 vessel, 2VO) plus 5% isoflurane-induced hypotension. **(C)** Representative image of fluorescent immunohistochemical (IHC) labeling for neurons (NeuN, green) in mouse hippocampus 7 days after global cerebral ischemia. Note post-injury reductions in NeuN+ cells in cornu ammonis-1 (CA1) sub-region (boxed). **(D,E)** Representative images of CA1 labeled for the astrocyte marker glial fibrillary acidic protein (GFAP, red) complexed with fluorescent *in situ* hybridization for miR-200c (green) expression. Note augmented miR-200c expression in astrocytes 7 days after global cerebral ischemia **(E)**.

**FIGURE 2:**
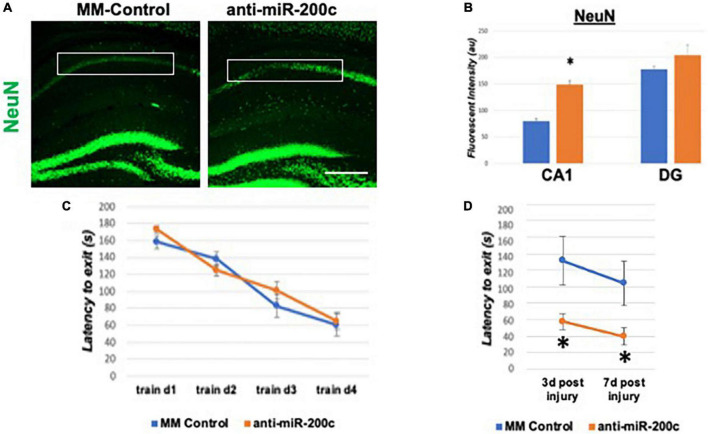
Effect of 2 h post-injury intravenous anti-miR-200c or MM-control treatment on sirtuin-1 (SIRT1) expression 7 days after global cerebral ischemia in mice. **(A)** Representative images of fluorescent IHC labeling for neurons (NeuN, green) in mouse hippocampus 7 days after global cerebral ischemia with and without post-injury anti-miR-200c treatment. **(B)** Quantification of post-injury CA1 NeuN fluorescence with and without IV anti-miR-200c post-treatment. **(C)** Escape latency during pre-injury Barnes maze training. **(D)** Escape latency at post-injury days 3 and 7 in mice with and without IV anti-miR-200c post-treatment. (*N* = 5–8 animals per treatment group, mean ± SEM, *p < 0.05). Scale bar, 1 mm.

A period of 10 min of 2VO and 5% isoflurane reliably resulted in hypotension (Figure 1A), cerebral hypoperfusion (Figure 1B) and in loss of CA1 neurons (Figure 1C). This injury paradigm also resulted in a significant (*p* < 0.05) increase by 9.8 + 4.5-fold in miR-200c expression in CA1, with visual evidence of post-injury augmentation of miR-200c expression in CA1 astrocytes *via* histological assessment (Figures 1D,E). Post-injury treatment with intravenous anti-miR-200c 2 h after global cerebral ischemia resulted in a significant (*p* < 0.05) reduction in post-injury CA1 miR-200c expression by 36.4 + 22% relative to animals treated with mismatch control sequence (MM-control), reduced post-injury weight loss by time-of-sacrifice (10.7 + 2 versus 19.2 + 6%) and improved overall survival (100 versus 72.5%). Anti-miR-200c treatment also significantly (*p* < 0.05) reduced loss of CA1 neurons at post-injury day 7 (Figures 2A,B). Conversely no differences were observed between treatments in the adjacent ischemia-resistant DG. Pre-injury Barnes maze training demonstrated progressive adaptive decreases in escape latency (Figure 2C). While global cerebral ischemia increased escape latency, post-injury IV treatment with anti-miR-200c significantly (*p* < 0.05) decreased escape latency at both post-injury days 3 and 7 (Figure 2D).

### Anti-microRNA-200c treatment increases global hippocampal mitochondrial dynamic protein expression after injury in a cell-type specific manner

**FIGURE 3:**
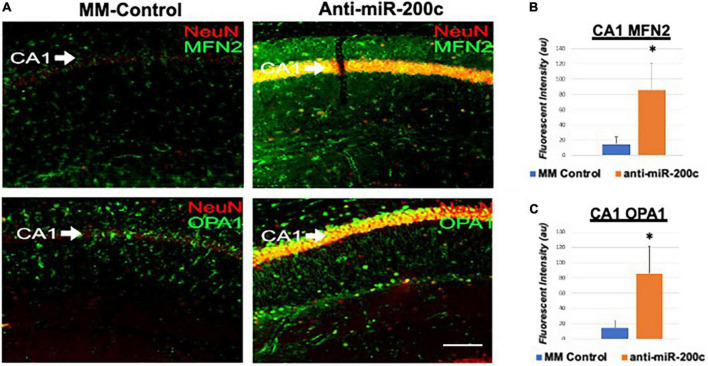
Mitofusin-2 (MFN2) and OPA1 expression in CA1 after global cerebral ischemia in mice. **(A)** Representative images of fluorescent IHC labeling of hippocampal NeuN (red) and MFN2 or OPA1 expression (green) 7 days after global cerebral ischemia with and without IV anti-miR-200c post-treatment. Quantification of MFN2 **(B)** and OPA1 **(C)** in all cells within CA1. *N* = 5–8 animals per treatment group, mean + SEM, *p < 0.05. Scale bar, 25 μm.

**FIGURE 4:**
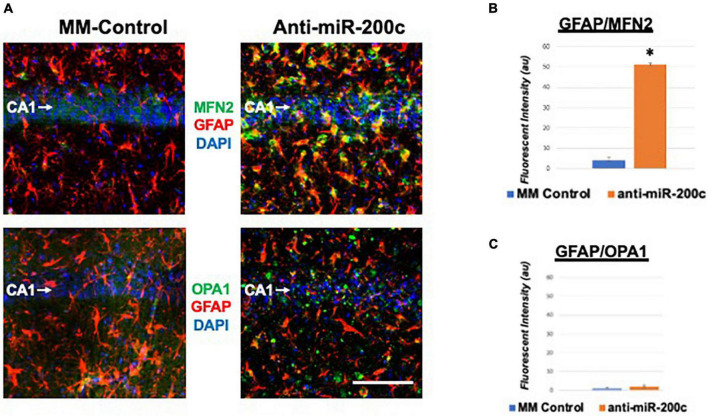
Astrocyte-specific CA1 MFN2 and OPA1 expression after global cerebral ischemia in mice. **(A)** Representative images of fluorescent IHC labeling of hippocampal GFAP (red) and MFN2 or OPA1 expression (green) 7 days after global cerebral ischemia with and without IV anti-miR-200c post-treatment. Quantification of co-labeled GFAP/mitofusin-2 (MFN2, **B**) and GFAP/OPA1 fluorescence **(C)** in CA1. *N* = 5–8 animals per treatment group, mean + SEM, *p < 0.05. Scale bar, 25 μm.

**FIGURE 5:**
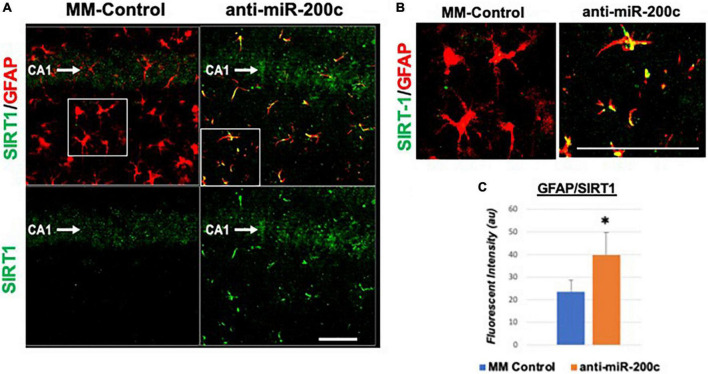
Astrocyte-specific CA1 SIRT1 expression after global cerebral ischemia in mice. Low power (200×, **A**) and high power (600×, **B**) representative images of fluorescent IHC labeling of hippocampal GFAP (red) and SIRT1 expression (green) 7 days after global cerebral ischemia with and without IV anti-miR-200c post-treatment. Quantification of co-labeled GFAP/SIRT1 **(C)** in CA1. *N* = 5–8 animals per treatment group, mean + SEM, *p < 0.05. Scale bar, 25 μm.

Next, we assessed hippocampal sub-regional expression of the mitochondrial fission proteins MFN2 and OPA1 in the CA1 and DG. Relative to DG, MFN2 expression significantly (*p* < 0.05) decreased in CA1 after global cerebral ischemia in mice treated with post-injury MM-control (Figures 3A,B). In contrast, mice treated with post-injury anti-miR-200c demonstrated significantly (*p* < 0.05) preserved CA1 MFN2 expression (Figures 3A,B). In parallel relative to DG, OPA1 expression significantly (*p* < 0.05) decreased in CA1 after global cerebral ischemia in mice treated with post-injury MM-control (Figures 3A,C), while mice treated with anti-miR-200c demonstrated significantly (*p* < 0.05) preserved CA1 OPA1 expression (Figures 3A,C). Cell-type specific analysis revealed that post-injury astrocyte-specific expression of MFN2 was significantly (*p* < 0.05) higher in mice treated with anti-miR-200c relative to MM-control treated mice (Figures 4A,B). Conversely no differences were observed in astrocyte-specific OPA1 expression between treatment groups, suggesting the global CA1 decrease in OPA1 occurred in alternative cell types (Figures 4A,C). Cell-type specific analysis of SIRT1 expression revealed significantly (*p* < 0.05) augmented post-injury expression isolated to CA1 astrocytes (Figures 5A–C).

### MicroRNA-200c modulates astrocyte oxidative stress *via* sirtuin-1

**FIGURE 6:**
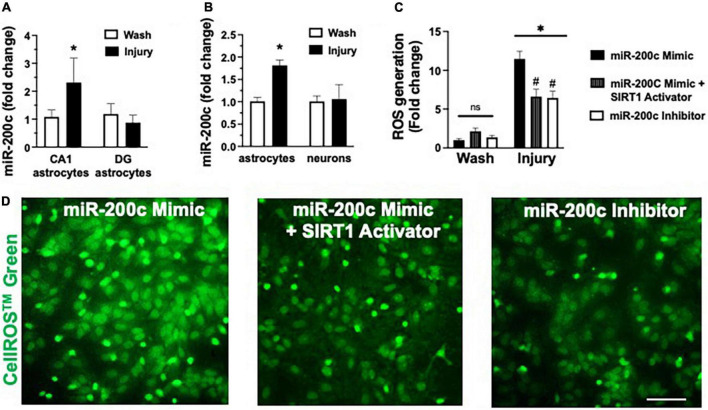
Post-injury miR-200c expression and SIRT1 activation and reactive oxygen species (ROS) production in astrocyte cultures. **(A)** Quantification of miR-200c expression after 3 h combined oxygen glucose deprivation (OGD) injury in primary hippocampal CA1 and DG astrocyte cultures. **(B)** Quantification of miR-200c expression after 3 h OGD in primary cortical astrocyte and neuronal cultures. **(C)** Quantification of ROS production in primary astrocytes pre-treated with MM-control, miR-200c mimic, miR-200c mimic + SIRT1 activator YK 3-237, or miR-200c inhibitor 24 h after 3 h OGD injury. **(D)** Representative live-cell images of astrocyte ROS production *via* CellROS™ Green fluorescence. *N* = 3 independent cultures, mean + SEM, *p < 0.05 versus within treatment wash control and ^#^*p* < 0.05 versus mimic injury. Scale bar, 25 μm.

*In vivo* we assessed miR-200c expression after 3 h OGD injury in primary hippocampal astrocyte cultures from CA1 and DG (Figure 6A), and in primary cortical astrocyte and primary cortical neuron cell cultures (Figure 6B). We observed a significant (*p* < 0.05) increase in miR-200c expression in both CA1 and primary cortical astrocyte cultures, but not in DG astrocyte or neuronal cultures (Figures 6A,B). To assess the role of miR-200c in oxidative stress, primary astrocyte cultures were pre-treated with either miR-200c mimic, mimic + 5 the SIRT1 activator YK 3-237 inhibitor 24 h prior to 24 h of glucose deprivation. Transfection by mimic significantly (*p* < 0.05) increased (157 + 22%) and inhibitor decreased (18 + 4%) miR-200c expression. Relative to MM-control sequence, miR-200c mimic significantly (*p* < 0.05) exacerbated ROS generation, an effect that was significantly attenuated by co-treatment with SIRT1 activator, while treatment with miR-200c inhibitor provided a comparable significant antioxidant effect (Figures 6C,D).

### MicroRNA-200c modulates astrocyte mitofusin-2 expression *via* sirtuin-1

**FIGURE 7:**
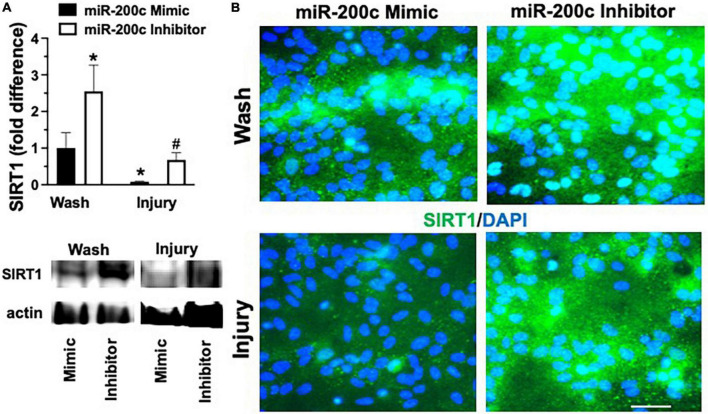
Effect of miR-200c on pre- and post-injury astrocyte SIRT1 in astrocyte cultures. Quantification **(A)** and representative images **(B)** of SIRT1 fluorescence 24 h after 3 h combined oxygen glucose deprivation injury in primary cortical astrocyte cultures with miR-200c mimic or miR-200c inhibitor pre-treatment. *N* = 3 independent cultures, mean + SEM, < 0.05 versus mimic wash control and ^#^*p* < 0.05 versus wash within treatment. Scale bar, 15 μm.

**FIGURE 8:**
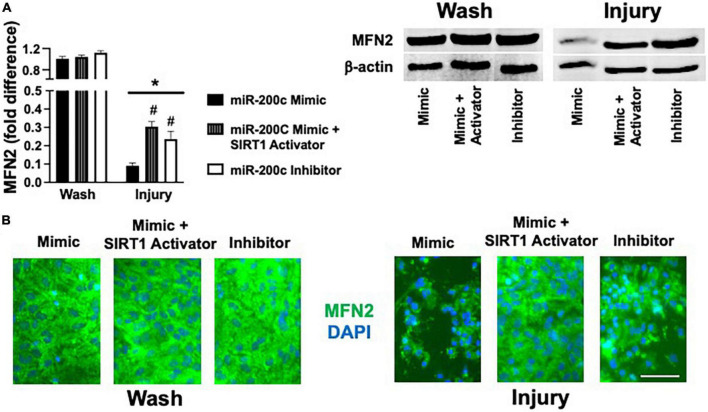
Effect of miR-200c and SIRT1 activation on pre- and post-injury astrocyte MFN2 expression in astrocyte cultures. Quantification **(A)** and representative images **(B)** of MFN2 fluorescence 24 h after 3 h combined oxygen glucose deprivation injury in primary cortical astrocyte cultures with miR-200c mimic, miR-200c mimic + SIRT1 activator YK 3-237, or miR-200c inhibitor pre-treatment. *N* = 3 independent cultures, mean + SEM, *p < 0.05 versus within treatment wash control and ^#^*p* < 0.05 versus mimic injury. Scale bar, 15 μm.

Primary astrocyte cultures were assessed for post-injury expression of SIRT1 and MFN2 with miR-200c mimic or inhibitor pre-treatment. Relative to miR-200c mimic, treatment with miR-200c inhibitor significantly increased SIRT1 expression in the absence of injury (Figures 7A,B). Twenty-four hours after 3 h OGD, SIRT1 expression was significantly (*p* < 0.05) decreased in both treatment groups relative to wash control, however, post-injury SIRT1 expression was significantly (*p* < 0.05) higher in miR-200c inhibitor-treated cells versus post-injury miR-200c mimic-treated cells (Figures 7A,B). In parallel OGD induced a significant (*p* < 0.05) reduction in MFN2 post-injury expression in primary cortical astrocyte cultures 24 h after 3 h OGD (Figures 8A,B). Similar to SIRT1, miR-200c inhibitor resulted in significantly preserved post-injury MFN2 expression in miR-200c inhibitor-treated cells, and in cells co-treated with miR-200c mimic and SIRT1 activator, relative to cells pre-treated with miR-200c mimic alone (Figures 8A,B).

## Discussion

Hippocampal CA1 circuits are central for memory formation (De Jong et al., 1999; Liu et al., 2005; Farkas et al., 2006; Bartsch et al., 2011; Lana et al., 2020). Clinical case studies of memory impairment in survivors of cardiac arrest have demonstrated that select loss of hippocampal CA1 neurons is the reason for their cognitive impairment (Zola-Morgan et al., 1986; Petito et al., 1987; Ng et al., 1989). This phenomenon has been repeatedly recapitulated in experimental rodent models of global cerebral ischemia (Kadar et al., 1998; Kristian and Hu, 2013), allowing for a stable platform for testing of therapeutic interventions to prevent CA1 neuronal loss and preserve CA1-dependent behavior. Cardiac arrest and resuscitation represent the largest clinical etiology of clinical transient global cerebral ischemia, with treatment options currently limited to mild hypothermia in survivors who have sustained severe neurological deficits. In the present study we observed neuroprotection in delayed CA1 neuronal cell death and reduced impairment in memory tasks with post-injury intravenous anti-miR-200c treatment after experimental global cerebral ischemia, demonstrating clinical efficacy for neuroprotection with a novel pharmaceutical approach within a clinically relevant 2 h post-injury treatment window.

MicroRNAs are central regulators of mitochondrial function, redox state, and apoptotic pathways (Ouyang et al., 2013a; Stary and Giffard, 2015). MiR-200c is highly enriched in the brain and is strongly induced by oxidative stress (Magenta et al., 2011; Stary et al., 2015; Arvola et al., 2021), augmenting ROS production in a positive feedback loop to dysregulate mitochondrial function (Carlomosti et al., 2017). We have previously demonstrated that pre-treatment with anti-miR-200c was protective in experimental stroke (Stary et al., 2015). Our observations in the present study indicate that post-injury anti-miR-200c therapy coincided with robust CA1 expression of MFN2. Metabolic energy state is closely associated with the balance between mitochondrial fusion and fission (Zhou et al., 2021), and mitochondria maintain a steady state of continuous fusion/fission maintaining the normal physiological function of cells (Dorn and Kitsis, 2015). Imbalance in mitochondrial dynamics can affect energy metabolism and post-stroke neuronal function by regulating the number, morphology, and function of mitochondria. Ischemia disrupts the dynamic balance of fusion and fission in part by inhibiting expression of fusion proteins MFN2 and OPA1 and recent studies in experimental stroke suggests that inhibition of mitochondrial fission and promotion of fusion is protective (Zhou et al., 2021).

Our observations from the present study indicate that augmented expression of CA1 MFN2 was localized to CA1 astrocytes, and *in vitro*, an early (3 h) increase in miR-200c in response to simulated ischemia was limited to CA1 and cortical astrocyte cultures. As specialized glia, astrocytes represent the most plentiful cell type in the mammalian brain, serving many housekeeping functions (Verkhratsky and Nedergaard, 2018) and indispensable for neurotransmitter homeostasis and maintenance and maturation of synapses (Pfrieger, 2009; Heneka et al., 2010). Neurons and astrocytes are functionally tightly coupled in the brain. Neurons consume 75–80% of total brain energy (Hyder et al., 2013) to support restoration of neuronal membrane potentials, for neurotransmitter synthesis, vesicle packaging, axoplasmic transport, and neurotransmitter release (Attwell and Laughlin, 2001; Rangaraju et al., 2014; Pathak et al., 2015). In astrocytes, energy stores are localized mainly as glycogen to buffer transient energy requirements from neurons (Kong et al., 2002) that do not store energy. Normal brain activity depends on metabolic plasticity of astrocytes and requires not only glucose supply from blood but also glycogen stored in astrocytes that can last beyond when glucose is depleted (Brown and Ransom, 2007). In response to inflammation or oxidative stress, astrocytes also upregulate glycolysis-producing ATP and lactate, which support energy metabolism to neurons (Voloboueva et al., 2008). Prior work has reported that selective dysfunction of hippocampal CA1 astrocytes occurs at early reperfusion times, long before CA1 neurons die (Ouyang et al., 2007) and that increased generation of ROS and mitochondrial dysfunction in CA1 astrocytes contribute to delayed death of CA1 neurons (Ouyang et al., 2007). Astrocytes in CA1 show early mitochondrial impairment and increased vulnerability to ischemia even when isolated in primary culture, compared to similarly isolated DG astrocytes. The results from the present study suggest that augmented CA1 astrocyte MFN2 may play a role in mitigating post-injury astrocyte mitochondrial destabilization, thereby leading to secondary CA1 neuronal protection.

Activation of SIRT1 has also been shown to be a promising therapeutic target for protective strategies for stroke (Zhu et al., 2010; Miao et al., 2016; Zhang et al., 2018). Oxidative stress induces regulatory changes in several SIRT1-associated genes, including those involved in metabolism, apoptosis, ion transport, cell motility, and G-protein signaling (Oberdoerffer et al., 2008). SIRT1 maintains bioenergetic balance by activating (PCG1-α) (Nogueiras et al., 2012), a transcriptional co-activator of respiratory genes and a regulator of mitochondrial biogenesis (Nemoto et al., 2005), and activates the transcription factor p53 which decreases ROS (Tan et al., 1999; Hussain et al., 2004; Sablina et al., 2005). SIRT1 can also exert *intercellular* control of mitochondrial function *via* glial cell line-derived neurotropic factor (GDNF) expression (Corpas et al., 2017), which functions to remotely maintain neuronal mitochondrial function (Mitroshina et al., 2019), and *via* p53, which is transported in extracellular exosomes (Pavlakis et al., 2020). SIRT1 is targeted by miR-200c: in brain microvascular endothelial cells miR-200c inhibition provided protection against *in vitro* ischemia *via* SIRT1 (Wang et al., 2019). In the present study SIRT1 activation was sufficient to mitigate the augmentation of ROS production with miR-200c mimic with ischemic injury in astrocytes, supporting a mechanistic role for miR-200c/SIRT1 in the present conditions. Prior studies have demonstrated SIRT1 preserves mitochondria after ischemia by selectively interacting with MFN2 (Biel et al., 2016). Our observations in the present study indicate that SIRT1 levels were augmented in CA1 astrocytes in mice and in primary astrocyte cultures with anti-miR-200c treatment, consistent with our observations identifying augmented MFN2 expression in astrocytes with post-injury anti-miR-200c treatment. Similar to oxidative stress, activation of SIRT1 in astrocytes was sufficient to reverse decreases in MFN2 after ischemic injury associated with miR-200c mimic, supporting a role for SIRT1 in mediating miR-200c regulation of astrocyte mitochondrial dynamics. Future studies should extend the mechanistic intercellular miR-200c/SIRT1/MFN2 signaling to the context of intercellular signaling between astrocytes and neurons, and as well extend preclinical testing of anti-miR-200c and SIRT1 activation as a post-injury therapy to include the biological variables of sex and age.

## Data availability statement

The raw data supporting the conclusions of this article will be made available by the authors, without undue reservation.

## Ethics statement

The animal study was reviewed and approved by the Stanford University Animal Care and Use Committee and in accordance with the National Institutes of Health Guide for the Care and Use of Laboratory Animals.

## Author contributions

BG and CS conceived the project and assembled and edited the manuscript. LX and XS performed experiments. BG, MG, and IM performed image and data analysis. All authors contributed to the article and approved the submitted version.
